# Practice recommendations for clinicians in ART centers in the context of COVID-19 pandemic: perspective of a developing country

**DOI:** 10.1186/s43043-020-00040-5

**Published:** 2020-09-17

**Authors:** Tarek A. Farghaly, Mohamed Fawzy, Ahmed M. Abbas

**Affiliations:** 1 Department of Obstetrics & Gynecology, Faculty of Medicine, Assiut University, Assiut, Egypt; 2 IVF Laboratory, IbnSina IVF Center, IbnSina Hospital, Sohag, Egypt; 3 Women Health Hospital, Assiut, 71511 Egypt

In December 2019, severe acute respiratory syndrome coronavirus 2 (SARS-CoV-2) firstly presented in a group of patients suffering from symptoms of pneumonia in Wuhan, China [1]. The disease caused by SARS-CoV-2 is coronavirus disease-2019 (COVID-19) that was considered as a worldwide pandemic by the World Health Organization, with more than 4,000,000 cases globally [2, 3].

In Egypt, in vitro fertilization (IVF) is a client-paid service with no medical insurance, the cost of IVF cycles is high relative to the average yearly income of Egyptians, and private centers mainly offer assisted reproduction services. Therefore, pragmatic solutions should be established to amend the recent guidelines to suit the local requirements in the context of the golden rule of “do no harm.” These solutions should be in conjunction with the latest guidelines to continue the proper management of patients while ensuring adequate resource allocation in response to COVID-19. We proposed the following measures adopted by an Egyptian IVF clinic to support the best practice and face the highly dynamic situation on day to day basis. This could provide guidance for low-resource countries that must cope with the pandemic for an extended period.

- Triaging women based on their symptoms (fever, cough) by phone call carried out by a medical intern prior to the scheduled visit to the ART center. For the new patients first seen in the center, triage is performed before access to the clinic for consultation by a medical intern wearing the appropriate personal protective equipment (PPE).
- Ensuring proper social distancing through proper respect of the time of scheduled visits with spacing out the appointment intervals between patients to minimize the crowding in the waiting area. Additionally, preventing any relative to the couple to enter the IVF center, especially children or adults over the age of 60 years.
- Laboratory evaluation of all patients before initiating the IVF cycle treatment through complete blood picture, C-reactive protein, erythrocyte sedimentation rate, and serum ferritin [4]. Those with suspicious results are excluded from initiating any cycle treatment. Given the limited data on when COVID-19 can be represented by blood level changes of the investigation mentioned above, we advise to repeat them once again on the day of ovulation trigger.
- Patients under immune suppression therapy and those who have medical disorders affecting the immune system are advised to postpone the IVF cycle treatment as the risk of acquiring the COVID-19 complications is high.
- Patients sign a written consent before starting the IVF cycle treatment, including the freeze-all embryos policy and deferring the transfer after the pandemic ends. Otherwise, if the couple insisted on fresh embryo transfer, we inform them that the available data on the effect of COVID-19 on pregnancy is limited [5].
- Using the fixed GnRH antagonist protocol and GnRH agonist triggering for all patients at risk of ovarian hyperstimulation syndrome (OHSS).
- Minimizing the monitoring visits by using the long-acting GnRH agonist drugs and long-acting gonadotropin stimulation drugs like corifollitropin alfa prefilled pin [6]. Additionally, we encourage the patients for self-administration of IVF medications at home to minimize contact with external personnel [7].
- The use of effective preoperative analgesia in patients requiring office hysteroscopy prior to IVF [8], and the use of paracervical block during the oocyte pick up to minimize the impact of anesthesia on respiratory function. Additionally, the use of airway intervention during general anesthesia leads to aerosol generation, which potentiates the risk of COVID-19 transmission to the surgical team [9].
- Patients who get pregnancy tested for B-HCG through providing a home blood sample by a laboratory technician and repeat it after 48 h to confirm doubling. Using Telehealth-based care through the Whatsapp messenger, follow-up of their condition and revising the medications in the first eight weeks at home is instituted.

As it is unclear how long the COVID-19 pandemic will continue, we are hoping that those measures could help the ART centers to provide their services for patients in developing countries, especially with low COVID-19 infection rates like Egypt. Moreover, the provided measures could be initially recommended in the centers located in countries with high infection rates such as the European Union and the USA after eradication of the COVID-19 pandemic.

## Data Availability

Not applicable.

## Abbreviations

ART: **A**ssisted reproductive technology
COVID-19: Coronavirus disease-2019
IVF: In vitro fertilization
OHSS: Ovarian hyperstimulation syndrome
PPE: Personal protective equipment
